# Supplementary material for: A consensus cell type atlas from multiple connectomes reveals principles of circuit stereotypy and variation
Source: bioRxiv. 2023 Jun 27:2023.06.27.546055. Preprint. [Version 1] doi: 10.1101/2023.06.27.546055 (PMC10327018; doi:10.1101/2023.06.27.546055)
Supplement: Supplement 8 [file NIHPP2023.06.27.546055v1-supplement-8.pdf]

bioRxiv preprint doi: https://doi.org/10.1101/2023.06.27.546055; this version posted June 27, 2023. The copyright holder for this preprint (which was not certified by peer review) is the author/funder, who has granted bioRxiv a license to display the preprint in perpetuity. It is made available under aCC-BY 4.0 International license.

Supplemental Figures

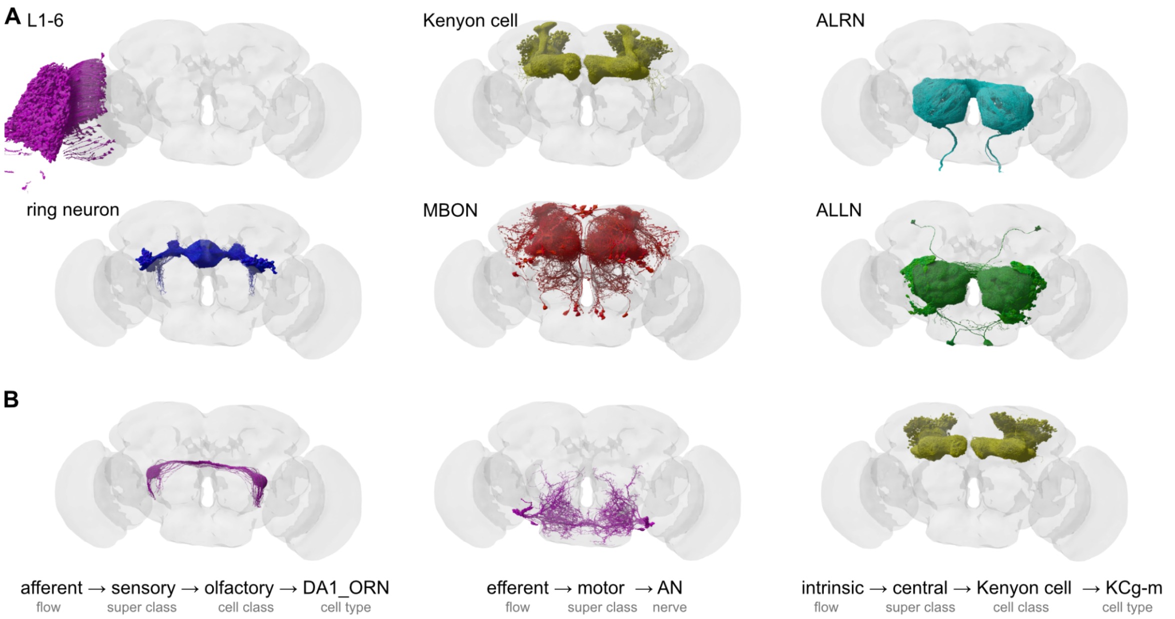

Supplemental Figure S1. A Examples for cell class annotations. **B** Examples for labels derived from the hierarchical annotations. Abbreviations: ALRN, antennal lobe receptor neuron; MBON, mushroom body output neuron; ALLN, antennal lobe local neuron; ORN, olfactory receptor neuron; AN, antennal nerve.

bioRxiv preprint doi: https://doi.org/10.1101/2023.06.27.546055; this version posted June 27, 2023. The copyright holder for this preprint (which was not certified by peer review) is the author/funder, who has granted bioRxiv a license to display the preprint in perpetuity. It is made available under aCC-BY 4.0 International license.

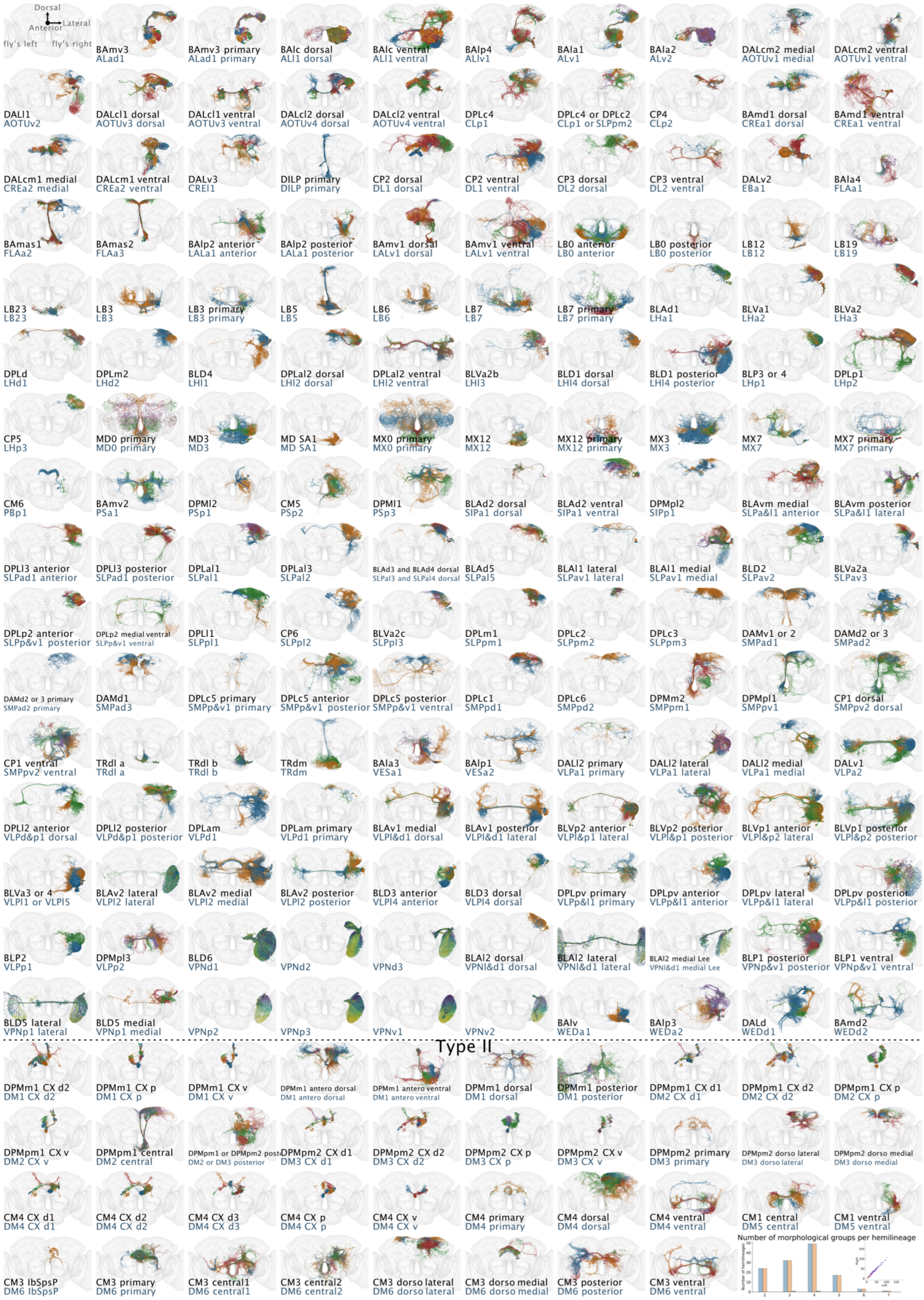

Supplemental Figure S2.1. Anterior views of neurons within a hemilineage (based on[53,102]), or neurons whose cell bodies form a cluster in a lineage clone (also referred to as "hemilineages" hereafter), based on the light-level data from[46–49,103]. The names of the hemilineages are at the bottom of each panel (top: Hartenstein nomenclature; bottom: ItoLee nomenclature). The snapshots only include neurons with cell bodies on the right

bioRxiv preprint doi: https://doi.org/10.1101/2023.06.27.546055; this version posted June 27, 2023. The copyright holder for this preprint (which was not certified by peer review) is the author/funder, who has granted bioRxiv a license to display the preprint in perpetuity. It is made available under aCC-BY 4.0 International license.

hemisphere, and the central unpaired lineages. Except for the hemilineages that tile the optic lobe, the neurons are coloured by morphological groups, obtained by the 'elbow method' (Methods, Hemilineage annotations section). The neurons that form cohesive tracts with their cell body fibres in the Type II lineages (see Methods) are at the lower part of the panels. The bottom right panel is a histogram of the number of morphological groups per hemilineage (blue: left; yellow: right; green: centre). Inset is the number of neurons per hemisphere for each morphological group, with points coloured by their density (yellow: denser). Corresponding group names, together with FlyWire and neuroglancer links are available in Supplementary Files 1 and 2.

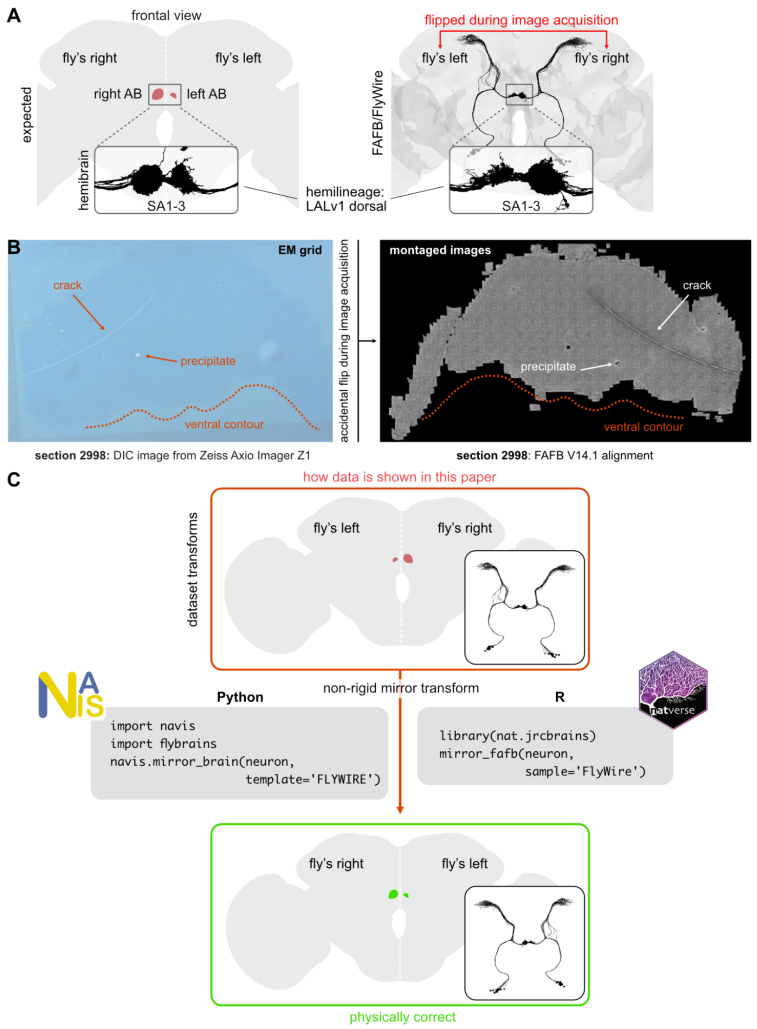

Supplemental Figure S2.2. A The asymmetric body (AB) is expected to be larger on the fly's right. The adult fly brain is conventionally shown in frontal views in 2D projections; this would place the fly's on the left of the page. In FAFB/FlyWire the situation initially appeared inverted. Insets show axons of SA1-3 neurons which form the major input to the AB. **B** Image of a brain section on the original EM grid (left) and the final image montage as shown in neuroglancer/CATMAID (right). Various landmarks are shown to illustrate the flip along the x-axis that explains the inversion. **C** Showcase of how to correct the inversion of FAFB/FlyWire data. For technical reasons, it was not possible to flip the whole FAFB volume and associated data. Therefore this must be corrected post hoc. Code samples show how this can be done for e.g. mesh or skeleton data using Python or R.

bioRxiv preprint doi: https://doi.org/10.1101/2023.06.27.546055; this version posted June 27, 2023. The copyright holder for this preprint (which was not certified by peer review) is the author/funder, who has granted bioRxiv a license to display the preprint in perpetuity. It is made available under aCC-BY 4.0 International license.

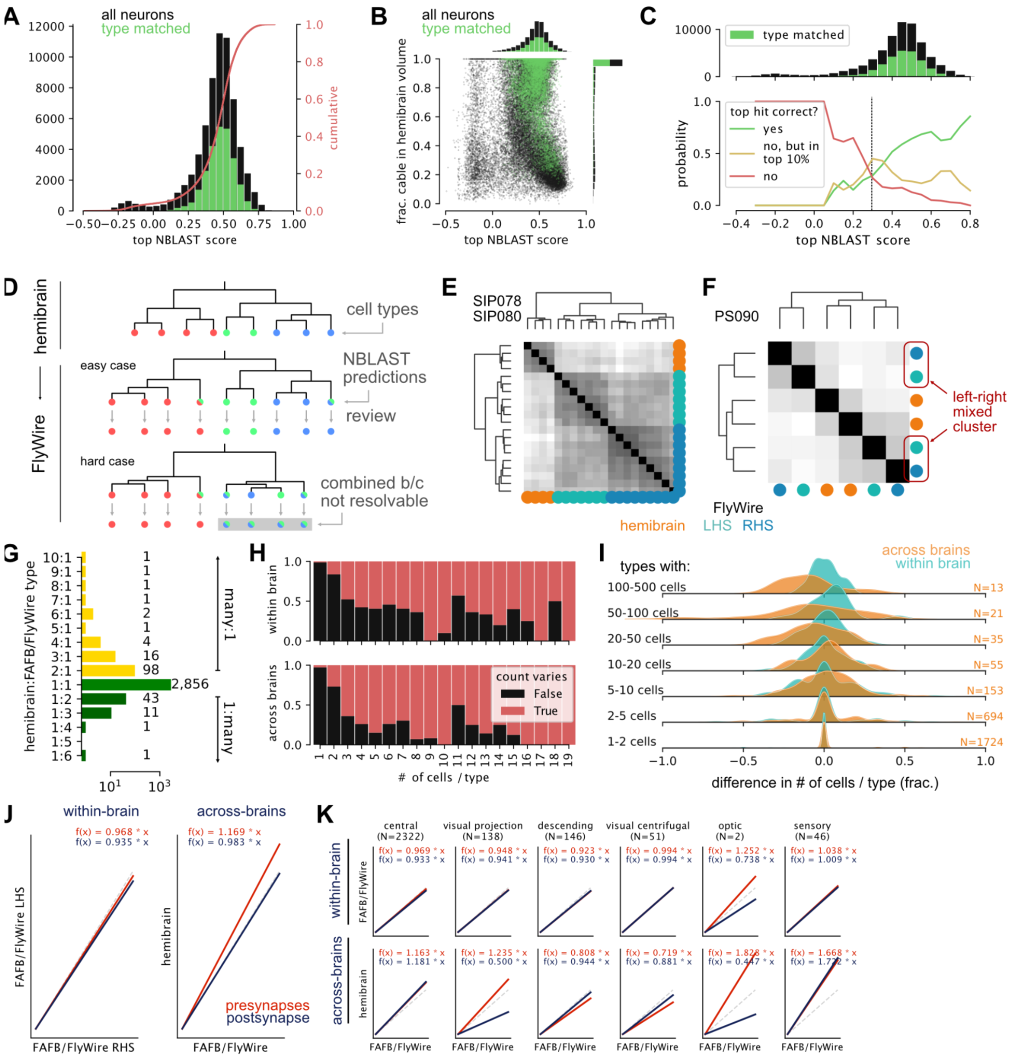

Supplemental Figure S3. **A** Distribution of top FlyWire → hemibrain NBLAST scores. **B** Top NBLAST scores vs fraction of neuron contained within hemibrain volume. Heavily truncated neurons typically produce bad scores. **C** Top: distribution of top NBLAST scores and fraction which was type matched. Bottom: probability that the correct hit was the top NBLAST hit (green) or at least among (yellow) the top 10% (by score) as a function of the top NBLAST score. **D** Explanation of match review process: where possible the within-dataset morphological clustering was taken into account. **E,F** Cross-brain co-clustering of the NBLAST scores for example cell types in Figure 3. **G** Counts for 1:many and many:1 type matches. **H** Fraction of cell types showing a difference in cell counts within (left/right, top) and across (bottom) brains. **I** Distribution of cell count differences. **J** Robust linear regression (Huber w/ intercept at 0) for within- and across-dataset pre/postsynapse counts from Figure 3H. **K** Same data as in J but separated by super class. Slopes are generally close to 1: 1.021 (pre-) and 1.035 (postynapses, i.e. inputs) between the left and right hemisphere of FlyWire, and 1.176 (presynapses, i.e. outputs) 0.983 (post) between FlyWire and the hemibrain. Note that correlation and slope are noticeably worse for cell types known to be truncated such as visual projection neurons which suggests that we did not fully compensate for the hemibrain's truncation and that the actual across-brain correlation might be even better.

bioRxiv preprint doi: https://doi.org/10.1101/2023.06.27.546055; this version posted June 27, 2023. The copyright holder for this preprint (which was not certified by peer review) is the author/funder, who has granted bioRxiv a license to display the preprint in perpetuity. It is made available under aCC-BY 4.0 International license.

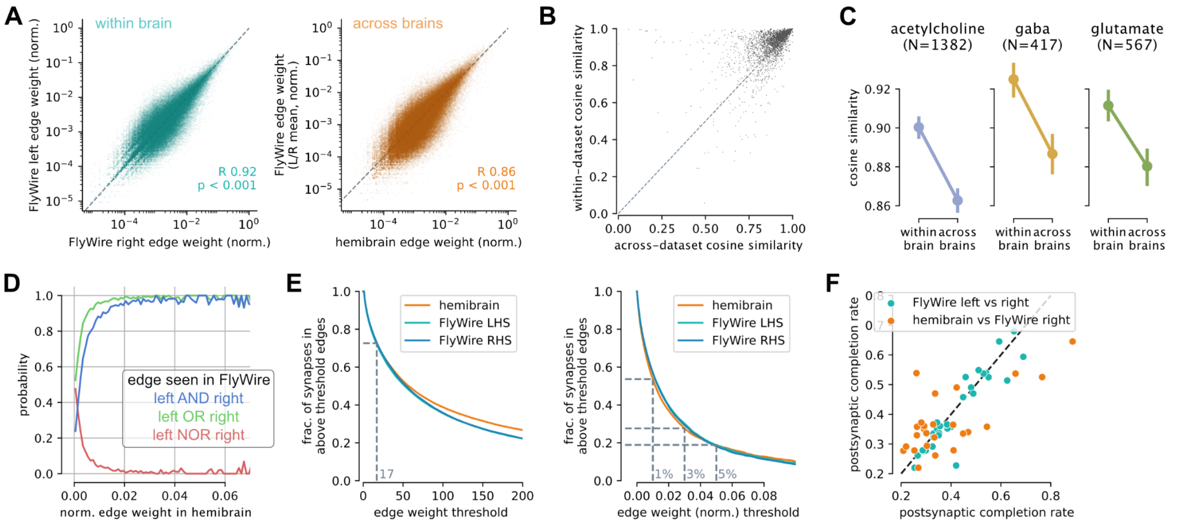

Supplemental Figure S4. A Comparison of normalised edge weights within (left) and across (right) brains. **B** Connectivity cosine connectivity similarity within and across brains. Each datapoint is a cell type identified across the three hemispheres. Size correlates with the number of cells per type. **C** Connectivity cosine similarity separated by neurotransmitter. **D** Probability that an edge present in the hemibrain is found in one, both or neither of the FlyWire hemispheres. **E** Fraction of synapses contained in edges above given absolute (left) and normalised (right) weight. **F** Postsynaptic completion rates. Each datapoint is a neuropil.

bioRxiv preprint doi: https://doi.org/10.1101/2023.06.27.546055; this version posted June 27, 2023. The copyright holder for this preprint (which was not certified by peer review) is the author/funder, who has granted bioRxiv a license to display the preprint in perpetuity. It is made available under aCC-BY 4.0 International license.

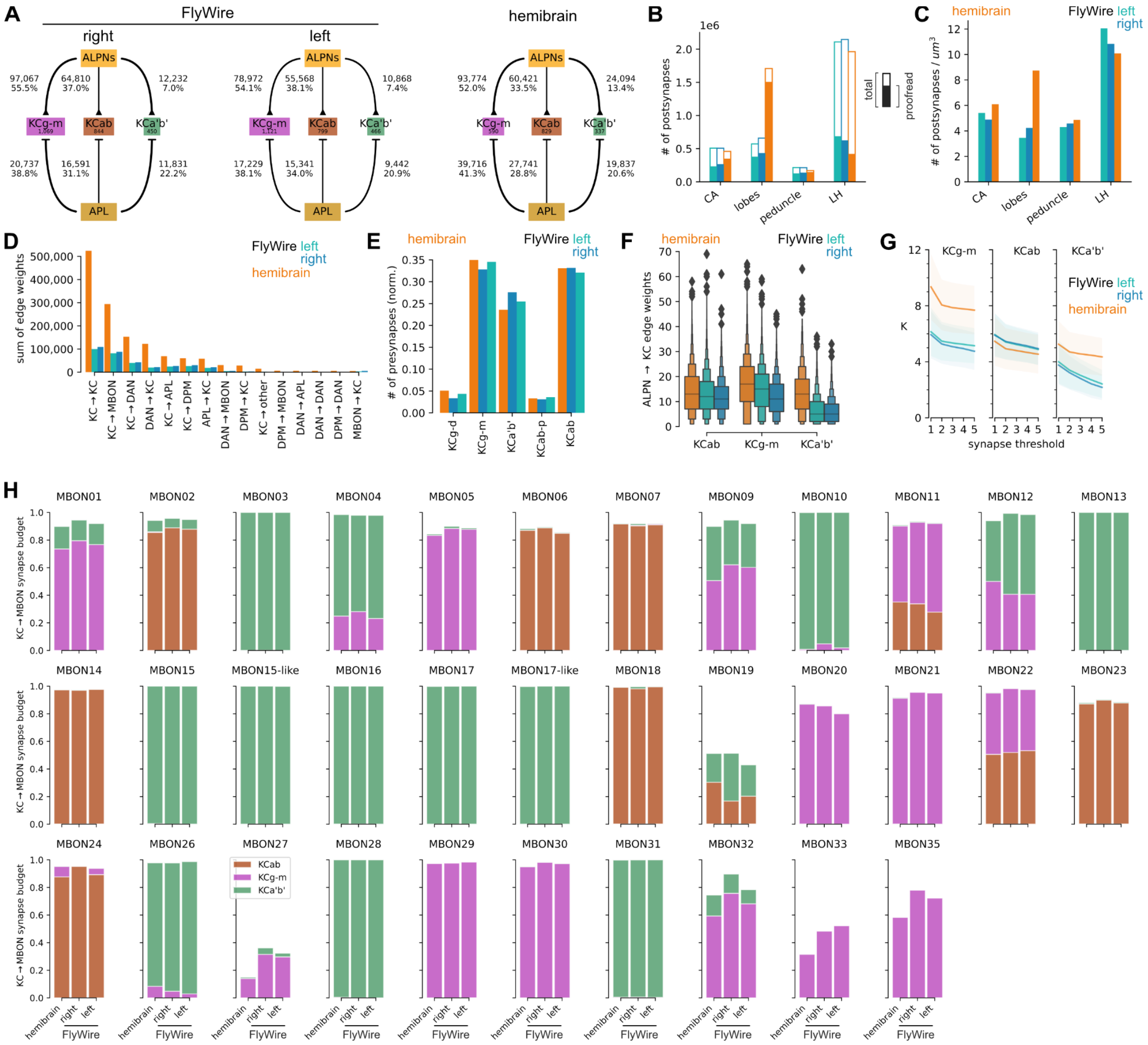

Supplemental Figure S5. **A** Graph showing ALPN/APL→KC connectivity across the three datasets. Edge labels give synapse counts and normalised to the total output budget of the source. In FlyWire, the mushroom bodies (MB) have 57.2% (left) and 60.7% (right) postsynaptic completion rate while the hemibrain MB has been proofread to 81.3% (see also B). To compensate for this we typically used normalised synapse counts and edge weights. Note that KCab act as an internal control as their numbers are consistent across all hemispheres and we don't expect to see any changes in their connectivity. **B** Total versus proofread postsynapse counts across MB compartments. Lateral horn (LH) shown for comparison. **C** Postsynapse density across MB compartments. **D** Sums of edge weights between different MB cell classes. **E** Presynapse counts per KC type normalised to the total number of KC synapses per dataset. **F** ALPN→KC edge weights. **G** *K* (# of ALPN types providing input to a single KC) under different synapse thresholds. **H** Fraction of MBON input budget coming from individual KCab, KCg-m and KCa'b'. Abbreviations: CA, calyx; DAN, dopaminergic neuron; ALPN, antennal lobe projection neuron; KC, Kenyon Cell; MBON, mushroom body output neurons.

bioRxiv preprint doi: https://doi.org/10.1101/2023.06.27.546055; this version posted June 27, 2023. The copyright holder for this preprint (which was not certified by peer review) is the author/funder, who has granted bioRxiv a license to display the preprint in perpetuity. It is made available under aCC-BY 4.0 International license.

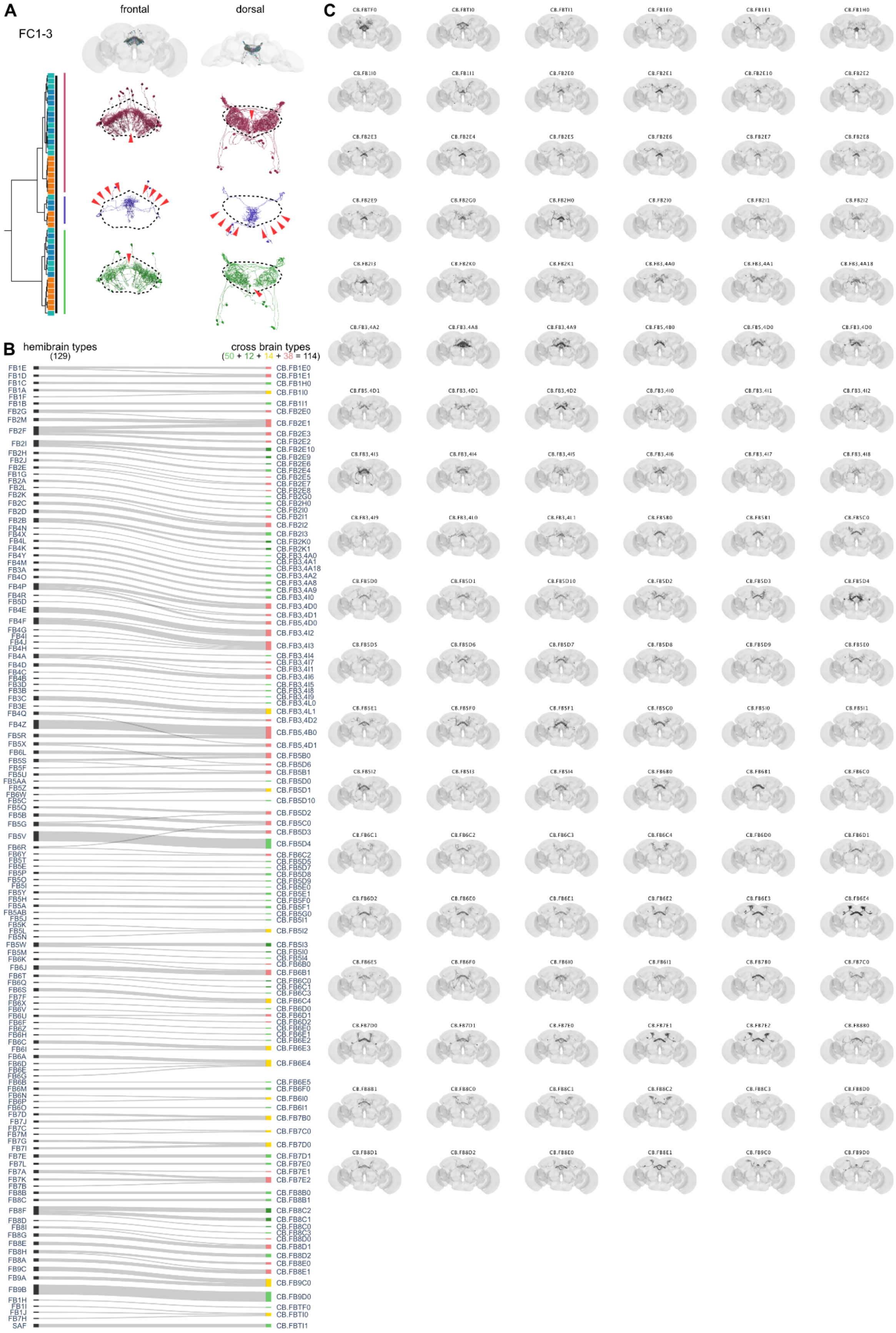

bioRxiv preprint doi: https://doi.org/10.1101/2023.06.27.546055; this version posted June 27, 2023. The copyright holder for this preprint (which was not certified by peer review) is the author/funder, who has granted bioRxiv a license to display the preprint in perpetuity. It is made available under aCC-BY 4.0 International license.

Supplemental Figure S6: A FC1-3 across-brain cluster from Figure 6D (asterisk) that was manually adjusted. This group consists of three sub-clusters that technically fulfil our definition of cell type. They were merged, however, because they individually omit columns of the fan-shaped body (arrowheads) and are complementary to each other. **B** Flow chart comparing FB1-9 hemibrain and cross-brain cell types. Colours correspond to 1:1, 1:many, many:1 and many:many mappings between hemibrain and cross-brain cell types. **C** Renderings of all FB1-9 cross-brain cell types.

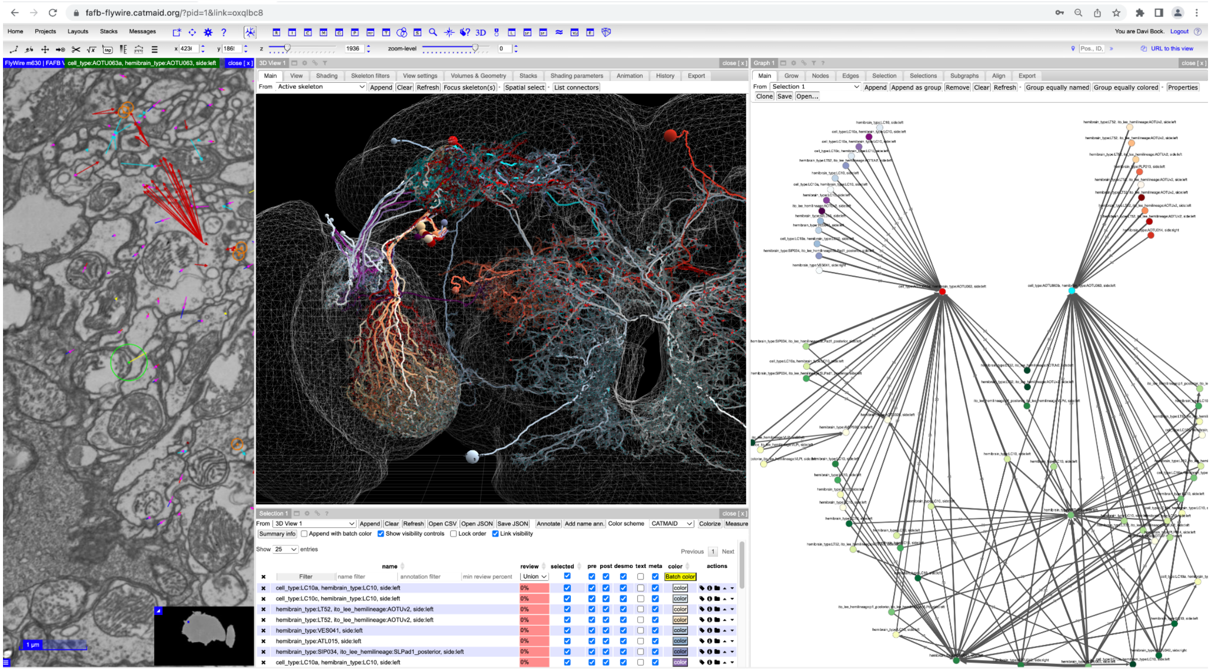

Supplemental Figure S7: Screenshot demonstrating the use of CATMAID Spaces (https://fafb-flywire.catmaid.org/) to interrogate the FlyWire connectome. Differential inputs to AOTU63a and b are visualised (red and cyan, respectively). The Graph widget was used to show all neurons making 20 or more synapses onto AOTU63a and b, and to show only >=20 synapse connections between these neurons. Neurons whose *only* >=20 synapse connection was to either AOTU63a or b (but not both) were differentially coloured (blue-purples and greens, respectively).

bioRxiv preprint doi: https://doi.org/10.1101/2023.06.27.546055; this version posted June 27, 2023. The copyright holder for this preprint (which was not certified by peer review) is the author/funder, who has granted bioRxiv a license to display the preprint in perpetuity. It is made available under aCC-BY 4.0 International license.

Supplemental Tables

flow	superclass	class	sub class	type	side	hemilineage	neurotransmitter
afferent	sensory	olfactory	ALRN	ORN_DA1	left	ALl1_ventral	acetylcholine
intrinsic	central	DAN		PAM08	left	CREa1_dorsal	dopamine
efferent	descending			DNa02	right	WEDd1	acetylcholine

Supplementary Table S1: Example annotations.

Field	Value	Ontology ID	Definition
flow	intrinsic		Neurons fully contained within the brain.
	afferent		Neurons that enter the brain from the periphery or the ventral nerve cord (VNC).
	efferent		Neurons that leave the brain towards the periphery or the VNC.
superclass	central	FBbt_00059245	Neurons fully contained within the central brain.
	ascending	FBbt_00048301	Neurons entering the brain from the VNC. These can be sensory or interneurons.
	descending	FBbt_00047511	Neurons with soma in the brain that exit the brain towards the VNC.
	endocrine	FBbt_00059246	Neurons that exit the brain via the NCC towards the ring gland.
	motor	FBbt_00005123	Neurons that exit the brain towards the periphery (and are hence assumed to be motor neurons).
	optic	FBbt_00007577	Neurons fully contained within the optic lobes or the ocellar ganglion. Includes some bilateral neurons (see class field).
	visual projection	FBbt_00048287	Neurons that have dendrites in the optic lobes or the ocellar ganglion and axons in the central brain.
	visual centrifugal	FBbt_00059244	Neurons that have dendrites in the central brain and axons in the optic lobes or the ocellar ganglion.
	sensory	FBbt_00005124	Neurons that enter the brain from the periphery. Note that “ascending” also includes some sensory neurons that we are unable to distinguish from ascending interneurons with any certainty.

Supplementary Table S2: Glossary for terms used in the top-most layers of the annotation hierarchy. Ontology ID refers to the Virtual Fly Brain database.

bioRxiv preprint doi: https://doi.org/10.1101/2023.06.27.546055; this version posted June 27, 2023. The copyright holder for this preprint (which was not certified by peer review) is the author/funder, who has granted bioRxiv a license to display the preprint in perpetuity. It is made available under aCC-BY 4.0 International license.

Class	Subset of superclass	Definition
bilateral	optic	Optic lobe neurons with projections into the contralateral optic lobes. Sometimes also leave synapses in the central brain.
ocellar	visual centrifugal	Projection neurons with dendrites in the brain and axons in the ocellar ganglia.
	visual projection	Projection neurons with dendrites in the ocellar ganglia and axons in the brain.
	optic	Neurons intrinsic to the ocellar ganglia. Cell bodies can be inside the brain though.
ALIN	central	“Antennal lobe input neurons”: neurons with dendrites out- and axons inside the antennal lobes. Does not include sensory neurons.
ALPN	central	“Antennal lobe projection neurons”: neurons with dendrites in the antennal lobe and axonal projections into the protocerebrum, lateral horn or calyx.
ALON	central	“Antennal lobe output neurons”: neurons with dendrites in the antennal lobes and axonal projections outside of the brain that are not canonical ALPNs.
ALLN	central	“Antennal lobe local neurons”: neurons with both dendrites and axons contained to the antennal lobes. Can be bilateral.
CX	central	Central complex neurons as defined by Hulse *et al.*[61]
DAN	central	Dopaminergic neurons (PAM and PPL) whose axons target the mushroom body lobes.
Kenyon Cell	central	Neurons with dendrites in the mushroom body calyx whose axons form the parallel fibre system of the mushroom body lobes.
MBIN	central	“Mushroom body input neuron”: APL or DPM.
LHCENT	central	“Lateral horn centrifugal neurons”: neurons with dendrites in the protocerebrum and axons in the lateral horn.
LHLN	central	“Lateral horn local neurons”: neurons with both dendrites and axons contained to the lateral horn.

Supplementary Table S3: Selection of cell *classes*.

Supplemental Files

Supplemental File 1 - Annotations

This TSV file contains all annotations including superclass, cell class, cell type, hemilineage, side, neurotransmitter and representative coordinates.

Supplemental File 2 - Summary with NGL links

This CSV file contains a summary of all hemilineages including neuroglancer links for viewing.

bioRxiv preprint doi: https://doi.org/10.1101/2023.06.27.546055; this version posted June 27, 2023. The copyright holder for this preprint (which was not certified by peer review) is the author/funder, who has granted bioRxiv a license to display the preprint in perpetuity. It is made available under aCC-BY 4.0 International license.

Supplemental File 3 - Hemilineage clustering

This CSV file contains details on the clustering of hemilineages into morphology groups.

Supplemental File 4 - Hemibrain metadata

This CSV file contains metadata for hemibrain neurons including columns for soma *side* and *cell class*.

Supplemental Videos

Supplemental Video 1

3D rendering showing all FlyWire neurons.

Supplemental Video 2

3D rendering showing all FlyWire neurons by superclass.

Supplemental Video 3

Slideshow for morphology groups by hemi-lineage.
